# Biostratigraphic significance of calcareous dinocysts in the Tithonian of the Pieniny Klippen Belt (southern Poland)

**DOI:** 10.7717/peerj.20935

**Published:** 2026-03-20

**Authors:** Agnieszka Ciurej, Marta Bąk, Krzysztof Bąk, Anna Wolska

**Affiliations:** 1 Department of Biology and Earth Sciences, University of the National Education Commission, Krakow, Poland; 2 Faculty of Geology, Geophysics and Environmental Protection, AGH University of Krakow, Krakow, Poland; 3 Department of Biology and Earth Sciences, University of the National Education Commission, Krakow, Poland

**Keywords:** Tithonian, Branisko succession, Upszar Limestone Member, Pieniny Limestone Formation, Calcareous dinocysts, Zones, Bioevents

## Abstract

Biostratigraphic studies of pelagic Tithonian limestones in the Western Tethys are important for regional palaeoenvironmental reconstructions, particularly for determining the onset of the accumulation of Maiolica-type sediments which dominated Early Cretaceous sedimentation in most deep-marine basins. These Tithonian limestones are rich in diverse planktonic microfossils, which constitute the basis of their biostratigraphic framework. In the lower Tithonian, this role is played by calcareous dinocysts, the subject of the present study conducted in the Polish segment of the Pieniny Klippen Belt in the Western Carpathians. Numerous and taxonomically diverse assemblages of these microfossils have been documented within a succession of argillaceous, variegated limestones overlain by whitish Maiolica-type limestones, exposed in a natural outcrop along Szeligowy Creek near Czarny Dunajec. These assemblages include species with stratigraphic significance, particularly those distinguished by differing first appearance datums (FADs). However, our analyses reveal a pronounced diachroneity of these FADs within Western Tethyan assemblages, as demonstrated through correlation of their datum events with the geomagnetic polarity timescale (GTS 2020). This finding underscores the need for caution when attempting stratigraphic correlations between various regions based solely on dinocyst zonations. For this reason, the four biostratigraphic zones identified in our study were correlated with magnetozones exclusively on the basis of data derived from two successions deposited within the same marine basin, for which magnetostratigraphic analyses were carried out concurrently. Based on these correlations, we infer that the onset of Maiolica-type sedimentation in the Pieniny Basin occurred during the younger part of chron M21n, corresponding to the younger interval of the *Semiformiceras fallauxi* chron in GTS 2020.

## Introduction

Calcareous dinoflagellate cysts (calcareous dinocysts, c-dinocysts) are spherical microscopic structures/cysts (10–180 µm in size) composed of walls built of calcite crystals with varying orientations. They belong to the family Thoracosphaeraceae, classified within the order Peridiniales and the class Dinophyceae (Elbrachter et al., 2008). The dinoflagellates that produce these calcareous cysts are photosynthetic, planktonic organisms inhabiting marine water (Tangen et al., 1982; Montresor et al., 1994). They are known to have existed from the Late Triassic to the present (Janofske, 1992; Bown, 1998), with approximately 260 fossil taxa (morphotypes) described (*e.g.*, Borza, 1964; Nowak, 1974; Řehánek, 1982; Řehánek, 1992; Fensome et al., 1993; Höll, Zonneveld & Willems, 1998; Reháková, 2000a; Reháková, 2000b; Zonnevald, Brune & Willems, 2000; Fensome & Williams, 2004; Vink, Zonneveld & Willems, 2000; Ciurej & Bąk, 2021; Ciurej, 2023). The group is well documented from Jurassic and Cretaceous shelf and basinal deposits across many regions of the world (*e.g.*,  Keupp, 1991; Zügel, 1994; Dias-Brito, 2000; Wendler, Gräfe & Willems, 2002; Ivanova & Kietzmann, 2017; Reháková & Rožič, 2019; Ciurej, Dubicka & Poberezhskyy, 2023; Fąfara et al., 2023; Kietzmann & Encinas, 2025). Due to their broad palaeogeographic distribution and moderate resistance to diagenetic alteration, calcareous dinocysts are widely used in stratigraphic and palaeoenvironmental reconstructions (*e.g.*,  Řehánek & Cecca, 1993; Dias-Brito, 2000; Ciurej, Bąk & Bąk, 2017; Lakova et al., 2017; Kowal-Kasprzyk, 2018; Kietzmann & Scasso, 2020; Kietzmann, Llanos & Iovino, 2023). Numerous biozonations for the Late Jurassic–Early Cretaceous interval have been established in the realm of the former Western Tethys (*e.g.*, Nowak, 1968; Borza, 1969; Vašíček, Michalík & Reháková, 1994; Lakova, Stoykova & Ivanova, 1999; Reháková, 2000a; Reháková, 2000b; Pszczółkowski, 2009; Reháková et al., 2011; Matejová et al., 2022). Many species and biozones defined in the Tethyan region have also been identified in distant basins worldwide, including the margins of Gondwana (North Africa: Benzaggagh & Atrops, 1996; Benzaggagh et al., 2015; South America: Ivanova & Kietzmann, 2017; Ruffo Rey, Kietzmann & Bressan, 2018; Kietzmann, Iovino & Encinas, 2022; Kietzmann, Llanos & Iovino, 2023; Kietzmann & Encinas, 2025; Antarctica: Kietzmann & Scasso, 2020) and the Caribbean Province (Pszczółkowski & Myczyński, 2010; López-Martínez et al., 2013). Some have additionally been recorded from the Russian–Arctic region corresponding to the Boreal Sea (Vishnevskaya, 2017).

**Figure 1:**
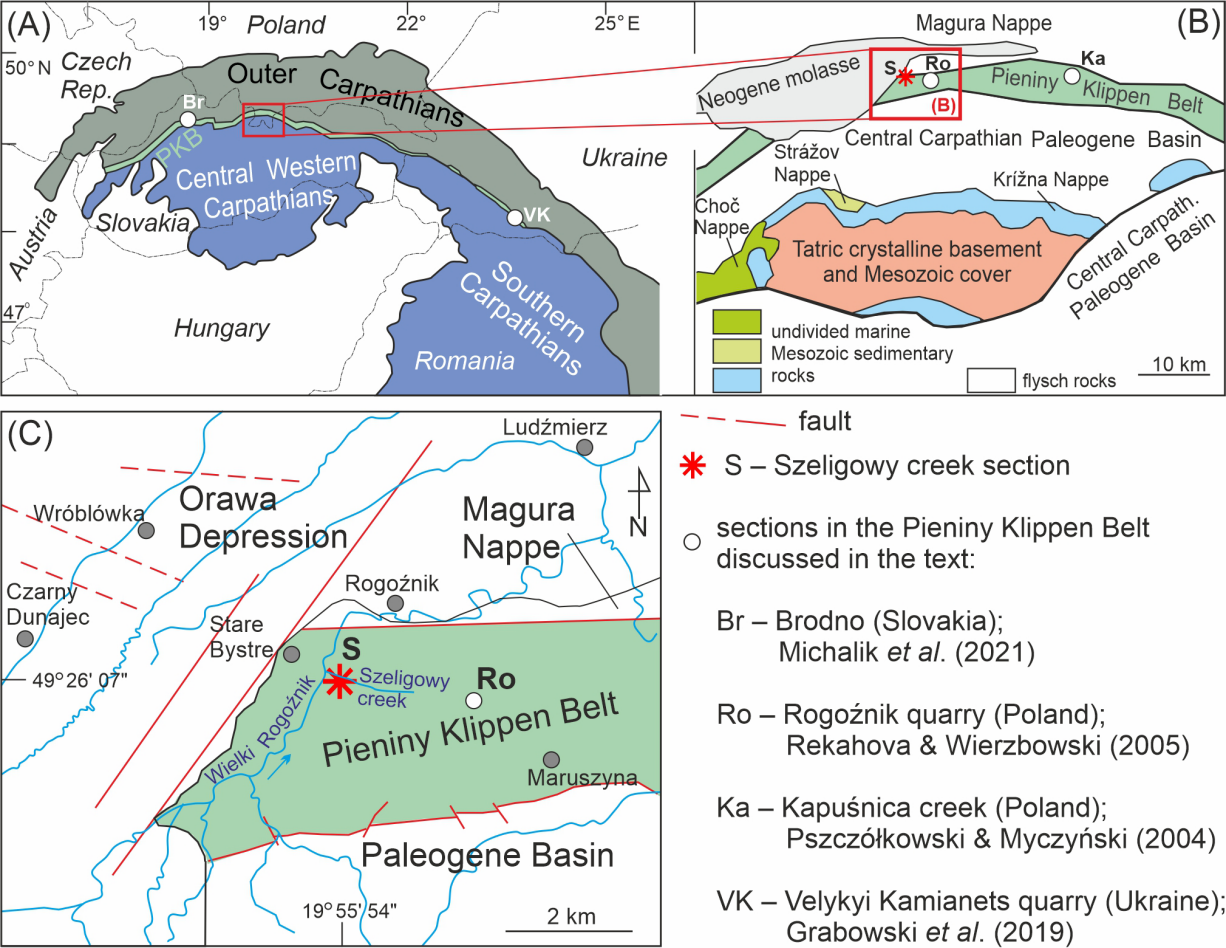
Geological position of the studied section (Szeligowy Creek) and discussed sections within the Pieniny Klippen Belt (PKB). (A) Sketch maps of the Carpathians with position of the PKB (Golonka & Picha, 2011). (B) Polish part of the Inner Carpathians and location of the studied area (modified after Birkenmajer, 1977; Golonka, 2011). (C) Simplified geological map of the PKB and its surroundings (modified after Prokešova, Plašienka & Milovský, 2012), with the detailed location of the studied section.

In this article, we present the biostratigraphy of the uppermost Jurassic (Tithonian) carbonate sediments of the Pieniny Klippen Belt (PKB) in the Western Carpathians (Fig. 1A), based on calcareous dinoflagellate cysts. These sediments were deposited in one of the deep basins of the Western Tethys. Samples for this study were collected from the Polish part of the PKB (Fig. 1B), within the Branisko tectonic-facies succession, in the natural outcrop along the Szeligowy Creek (Fig. 1C), which exposes a transition profile from radiolarites to a pelagic limestone succession, characteristic of many Oxfordian–Tithonian outcrops in various parts of the Alpides. We present the results of detailed taxonomic analyses of the calcareous dinoflagellate assemblages and define local biostratigraphic zones that we correlate with magnetostratigraphy. The correlation is preceded by a discussion of the ranges of selected index taxa from Tethyan profiles in relation to magnetozones, which, together with ammonite biostratigraphy, serve as the primary chronostratigraphic markers for this time interval (Hesselbo, Ogg & Ruhl, 2020).

## Geological Setting

**Figure 2:**
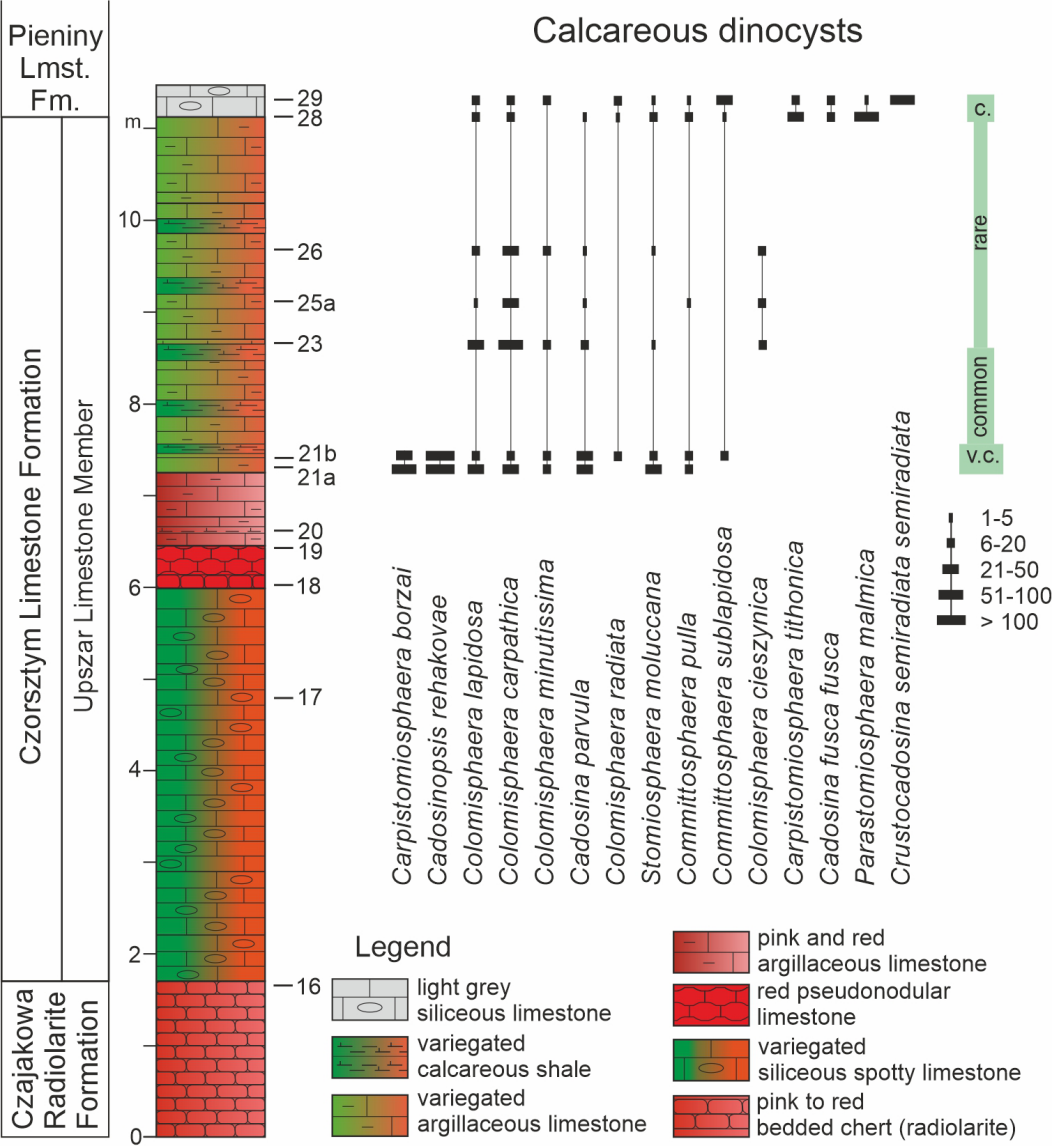
Lithological column of the Szeligowy Creek section, with distribution and relative abundance of calcareous dinocyst species. Lithostratigraphy after Birkenmajer (1977).

The Pieniny Klippen Belt (PKB) is a narrow (approximately 1–20 km wide) and long (approximately 600 km) geological unit located between the Inner and Outer Carpathians (*e.g.*, Birkenmajer, 1977; Figs. 1A, 1B). It is strongly folded and tectonised, with a considerable reduction in the space of the original sedimentary basins (*e.g.*, Birkenmajer, 1986; Golonka, 2011). This structural unit contains Mesozoic and Paleogene sedimentary rocks, involved in several tectonic-facies units, partly corresponding to the depth zones of the Pieniny Basin (*e.g.*, Birkenmajer, 1986). The sediments of one of these units, the Branisko succession, were deposited in the more distal areas of this basin, in the vicinity of the Pennine rift system, which was gradually occupied by the Pennine Ocean during the Middle Jurassic through the Cretaceous (*e.g.*, Michalík, 1994; Plašienka, 2003). During the Late Jurassic–Early Cretaceous, pelagic, deep-water sedimentation of siliceous and carbonate oozes dominated this basin (*e.g.*, Birkenmajer, 1953; Birkenmajer, 1977; Bąk, Bąk & Michalik, 2018). Currently, these sediments are represented by red radiolarites (the Czajakowa Radiolarite Formation; Fig. 2), reddish to greenish nodular limestones with intercalations of thin radiolarite layers (the Upszar Limestone Member of the Czorsztyn Limestone Formation), and whitish Maiolica-type limestones (the Pieniny Limestone Formation) (*e.g.*, Birkenmajer, 1977; Bąk et al., 2018).

Transition succession from siliceous to carbonate sediments (the Upszar Limestone Member; Branisko and Pieniny Units) and their counterparts in shallower facies (Coquina Limestone Member; Czorsztyn Unit) has been widely discussed in stratigraphic studies based on aptychi, calpionellids, calcareous dinocysts, calcareous nannoplankton, ammonites and radiolarians (Birkenmajer & Gąsiorowski, 1960; Birkenmajer & Gąsiorowski, 1961; Gąsiorowski, 1962; Myczyński, 1973; Nowak, 1976; Kutek & Wierzbowski, 1986; Pszczółkowski & Myczyński, 2004; Reháková & Wierzbowski, 2005; Reháková et al., 2011). The age of this unit was generally constrained as the Tithonian (Pszczółkowski & Myczyński, 2004) or late Kimmeridgian–Tithonian (Reháková & Wierzbowski, 2005). More recent studies conducted in the Slovak part of this unit (Michalík et al., 2016; Michalík et al., 2021), including magnetostratigraphic analyses, indicate that their accumulation terminated close to the lower boundary of the M19r magnetozone, which corresponds to the *Protacanthodiscus andraeai* ammonite zone according to the Geological Time Scale 2020 (Hesselbo, Ogg & Ruhl, 2020).

## Materials and Methods

The Szeligowy section (49°26′07″N, 19°55′54″E) is situated in the PKB in southern Poland along the Szeligowy Creek, which is the righthand tributary of the Wielki Rogoźnik River (Fig. 1C), near the town of Czarny Dunajec. The 20 m-thick section comprises the Czajakowa Radiolarite Formation, the Czorsztyn Limestone Formation (Upszar Limestone Member), and the lowermost part of the Pieniny Limestone Formation. Calcareous dinocysts, however, were found only in seven samples taken from a 10-meter interval of variegated argillaceous limestones belonging to the Upszar Limestone Member and the basinal portion of the Pieniny Limestone Formation (Fig. 2).

Calcareous dinocysts were studied from thin sections (3 × 5 cm), examined under a Nikon Eclipse LV100N POL polarizing optical microscope with a digital camera Nikon DS Ri2 and NIS-Elements BR software. To obtain optimal images of the dinocysts, various microscope settings were applied, including adjustments of illumination: (a) transmitted light (plane-polarized light), (b) crossed polarized light, (c) reduced condenser-stage distance, and (d) fully open or partially closed aperture diaphragm. The thin sections collection is housed in the first author’s repository (UPKG/2/2020) at the Department of Biology and Earth Sciences, University of the National Education Commission in Krakow, Poland.

## Results

### Calcareous dinocyst assemblages

Fifteen species of calcareous dinocysts belonging to eight genera were identified (Fig. 2). Species diversity within the assemblages is moderate, ranging from 5 to 11 species per sample. Specimen abundance is variable. The most abundant assemblages occur in the oldest sediments of the Upszar Limestone Member (Szel-21a and Szel-21b) and the Pieniny Limestone Formation (Szel-28, Szel-29). The state of preservation of the cysts is generally good to very good across most observation fields, despite the moderate recrystallization of the surrounding micrite. Only locally, where silicification is present, are the cysts poorly preserved, precluding their unambiguous identification. Such specimens are classified as problematic taxa.

**Figure 3:**
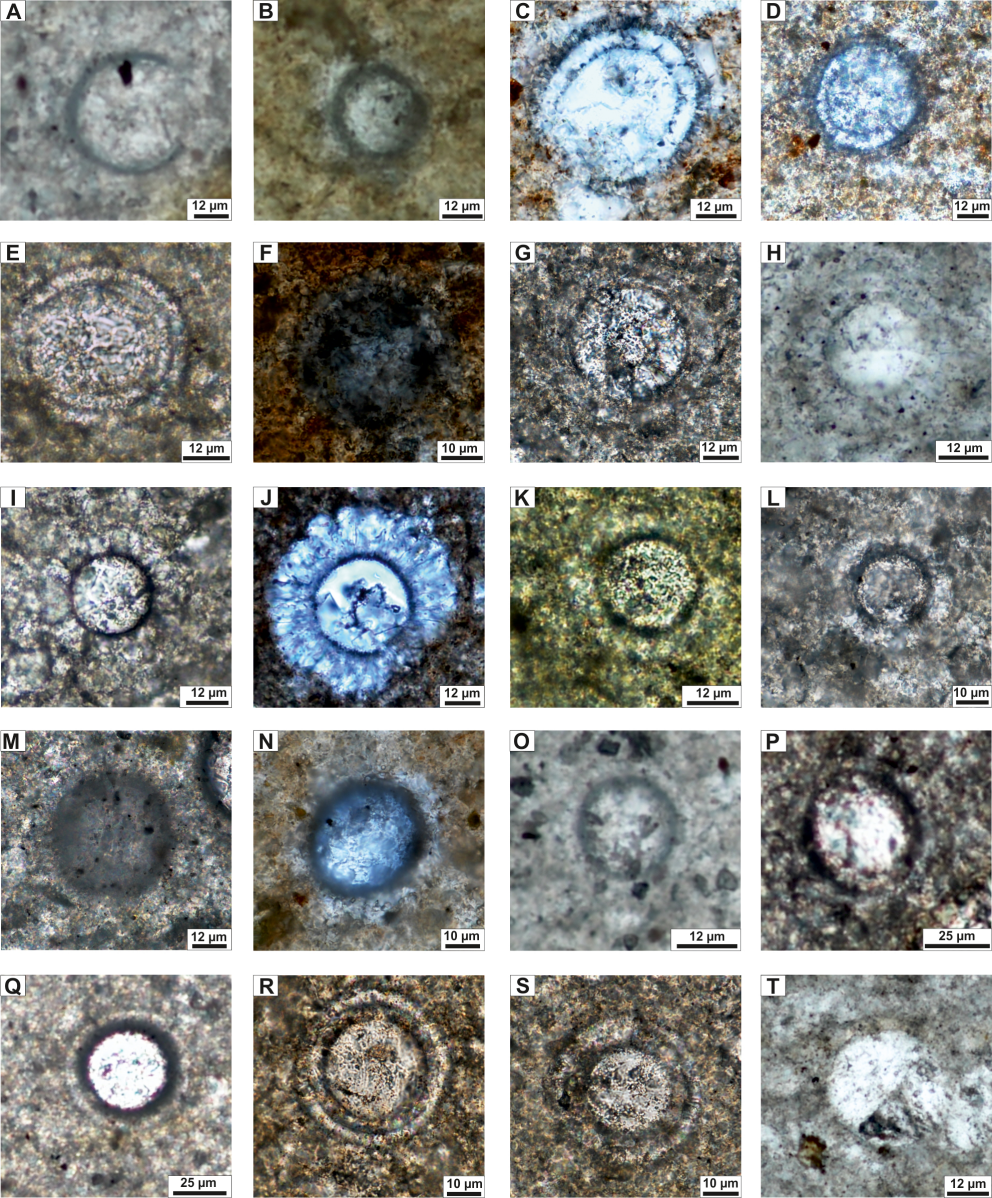
Calcareous dinoflagellate taxa from the Szeligowy Creek section of the Pieniny Klippen Belt. (A) *Cadosina fusca fusca* Wanner, sample Szel-29; (B) *Cadosina parvula* Nagy, sample Szel-21a; (C, D) *Cadosinopsis rehakovae* Ciurej & Bąk; (C) elongate sections, (D) transverse section, sample Szel-21b; (E, F) *Carpistomiosphaera borzai* (Nagy), sample Szel-21a; (G) *Carpistomiosphaera tithonica* Nowak, sample Szel-29; (H, I) *Colomisphaera carpathica* (Borza), (H) sample Szel-23, (I) sample Szel-25a; (J) *Colomisphaera cieszynica* Nowak, sample Szel-26; (K) *Colomisphaera lapidosa* (Vogler), sample Szel-28; (L) *Colomisphaera minutissima* (sensu Nowak), sample Szel-21a; (M) *Colomisphaera radiata* (Vogler), sample Szel-29; (N) *Committosphaera pulla* (Borza), sample Szel-21a; (O) *Committosphaera sublapidosa* (Vogler), sample Szel-29; (P, Q) *Crustocadosina semiradiata semiradiata* (Wanner), sample Szel-29; (R, S) *Parastomiosphaera malmica* (Borza), sample Szel-28; (T) *Stomiosphaera moluccana* Wanner, sample Szel-21a.

The assemblages of the Upszar Limestone Member (samples from Szel-21a to Szel-26) comprise a total of eleven species. Both the abundance and diversity of dinocysts decreases upward in the section. The oldest assemblages (Szel-21a, Szel-21b) are dominated by cysts of *Cadosinopsis rehakovae* Ciurej & Bąk (Figs. 3C, 3D) and *Carpistomiosphaera borzai* (Nagy) (Figs. 3E, 3F), accompanied by forms assigned to *Cadosina parvula* Nagy (Fig. 3B), *Colomisphaera carpathica* (Borza) (Figs. 3H, 3I), *Colomisphaera cieszynica* Nowak (Fig. 3J), *Colomisphaera lapidosa* (Vogler) (Fig. 3K), *Colomisphaera minutissima* (*sensu* Nowak), (Fig. 3L), *Colomisphaera radiata* (Vogler) (Fig. 3M), *Committosphaera pulla* (Borza) (Fig. 3N), *Committosphaera sublapidosa* (Vogler) (Fig. 3O), and *Stomiosphaera moluccana* Wanner (Fig. 3T).

This assemblage, with the exception of *C. borzai* and *C. rehakovae*, is also present in the overlying sediments of the Pieniny Limestone Formation, where specimens *Carpistomiosphaera tithonica* Nowak (Fig. 3G), *Cadosina fusca fusca* Wanner (Fig. 3A), *Parastomiosphaera malmica* (Borza) (Figs. 3R, 3S) and *Crustocadosina semiradiata semiradiata* (Wanner) (Figs. 3P, 3Q) appear for the first time and are found in high abundance.

## Discussion

### Comparison of Kimmeridgian and Tithonian calcareous dinocyst ranges from the Western Tethys

The taxonomic diversity of the dinocyst assemblages observed in the studied section (Fig. 2) is analysed here from a biostratigraphic perspective. Considering previous research on this group of microfossils from various pelagic facies of the Kimmeridgian–Berriasian interval in the PKB, it can be expected that they may document the Tithonian. This has been indirectly indicated by studies conducted in the Polish part of the Branisko succession of the PKB (Kapuśnica 1 section; Pszczółkowski & Myczyński, 2004) and in the Czorsztyn succession of the PKB (Rogoźnik quarry section; Reháková & Wierzbowski, 2005). In the Kapuśnica 1 profile, where seven dinocyst species were described (six of which also occur in the studied section), the stratigraphic position of the entire assemblage was determined based on the FO of *Calpionella alpina*, *i.e.,* below this event. The juvenile ammonite found there, identified as *Protancyloceras* sp., may additionally support the Tithonian age of these deposits (compare its ranges according to Wierzbowski, 1990, and Sarti, 1999).

In the Rogoźnik section, a rich assemblage of calcareous dinocysts (15 species, including 10 also present in the studied profile) identified in the Rogoża Coquina Member is interpreted as representing the Kimmeridgian and the lower part of the Tithonian, *i.e.,* below the first appearance of calpionellids (FO of *Longicollaria dobeni* (Borza); Reháková & Wierzbowski, 2005). Additional evidence for the Tithonian age in this section is provided by shells of several ammonite species collected from loose material (Kutek & Wierzbowski, 1986) and previously described from this locality, although without precise stratigraphic positioning (Birkenmajer, 1962; Birkenmajer, 1963; Birkenmajer, 1977). According to Reháková & Wierzbowski (2005), these ammonites indicate that the part of the section containing calcareous dinocysts can be generally correlated with Tithonian ammonite zones: *Hybonoticeras hybonotum* through *Semiformiceras semiforme*. In both profiles, the authors proposed a local dinocyst zonation, but the precise correlation of these zones with the chronostratigraphic scale was not possible. Marked differences in the first appearance datum (FAD) and last appearance datum (LAD) of the dinocyst taxa between the two sections are also evident.

For this reason, before defining local biostratigraphic horizons in the studied section, we first examined the ranges of calcareous dinocysts based on sedimentary profiles deposited exclusively within the Western Tethys. In addition to studies of calcareous dinocysts and calpionellids, magnetozone ranges were also analysed. For the Kimmeridgian and Tithonian, magnetostratigraphy represents the most reliable chronostratigraphic tool (see Hesselbo, Ogg & Ruhl, 2020), alongside ammonite zonation and zonation based on calcareous nannoplankton (the latter applying only to the younger part of the Tithonian).

**Figure 4:**
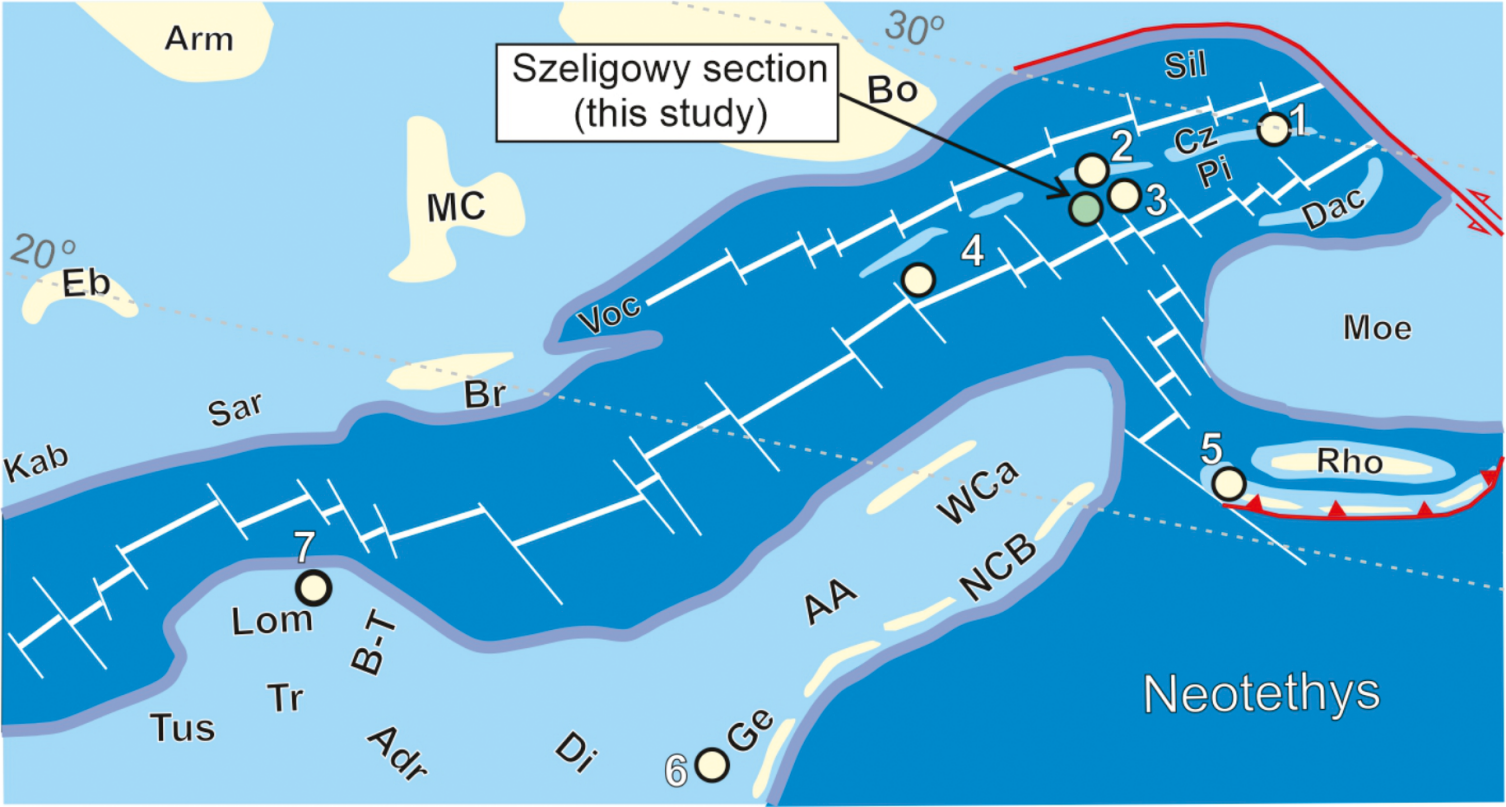
Palaeogeographic map of Alpine Tethys realm during the Berriasian (142 Ma) with the position of the section studied and sections discussed in the text; modified after Stampfli & Hochard (2009), Schmid et al. (2020), and Lodowski et al. (2024). Abbreviations of geographic regions: AA, Austro Alpine; Adr, Northern Adriatic Basin; Arm, Armorican Massif; Bo, Bohemian Massif; Br, Brianconnais; B-T, Belluno–Tarvisio Basin; Cz, Czorsztyn Ridge; Dac-Dacia; Di, Dinaric (Friulli) Platform; Eb, Ebro Massif; Ge, Gerecse Basin; Kab, Kabylies; Helv, Helvetic shelf; Lom, Lombardian Basin; MC, Massif Central; Moe, Moesian Platform; NCB, Neotethyan Collision Belt; Pi, Pieniny Basin; Rho, Rhodopes; Sar, Sardinia; SR, Silesian Ridge; Sil, Silesian Basin; Tr, Trento Platform; TrD, Transdanubian Range; Tus, Tuscan; UM, Umbria-Marche Platform; Voc, Vocontian Basin; WCa, Western Carpathians. Sections discussed in the text: 1–Velykyi Kamianets (Czorsztyn Ridge; Grabowski et al., 2019), 2–Rogoźnik (Czorsztyn Ridge; Kutek & Wierzbowski, 1986; Reháková & Wierzbowski, 2005), 3–Kapuśnica (Pieniny Basin; Pszczółkowski & Myczyński, 2004), 4–Brodno (Pieniny Basin; Michalík et al., 2021), 5–Barlya (Western Balkan Basin; Lakova et al., 2017), 6–Hárskút (Gerecse Basin; Lodowski et al., 2022), 7–Torre de’ Busi (Lombardian Basin; Petrova et al., 2025).

Two sections from the PKB were selected for this analysis: Velykyi Kamianets, Ukraine (Grabowski et al., 2019; former Czorsztyn Submarine Ridge; section 1 in Fig. 4) and Brodno, Slovakia (Michalík et al., 2021; former Pieniny Basin; section 4 in Fig. 4). Additionally, the dinocyst ranges from the Hárskút section, Hungary (Lodowski et al., 2022; former Gerecse Basin), Barlya section, Bulgaria (Lakova et al., 2017; former Western Balkan Basin), and Torre de’ Bussi section, Italy (Petrova et al., 2025; former Lombardian Basin) were incorporated into the comparison (see Fig. 4).

**Figure 5:**
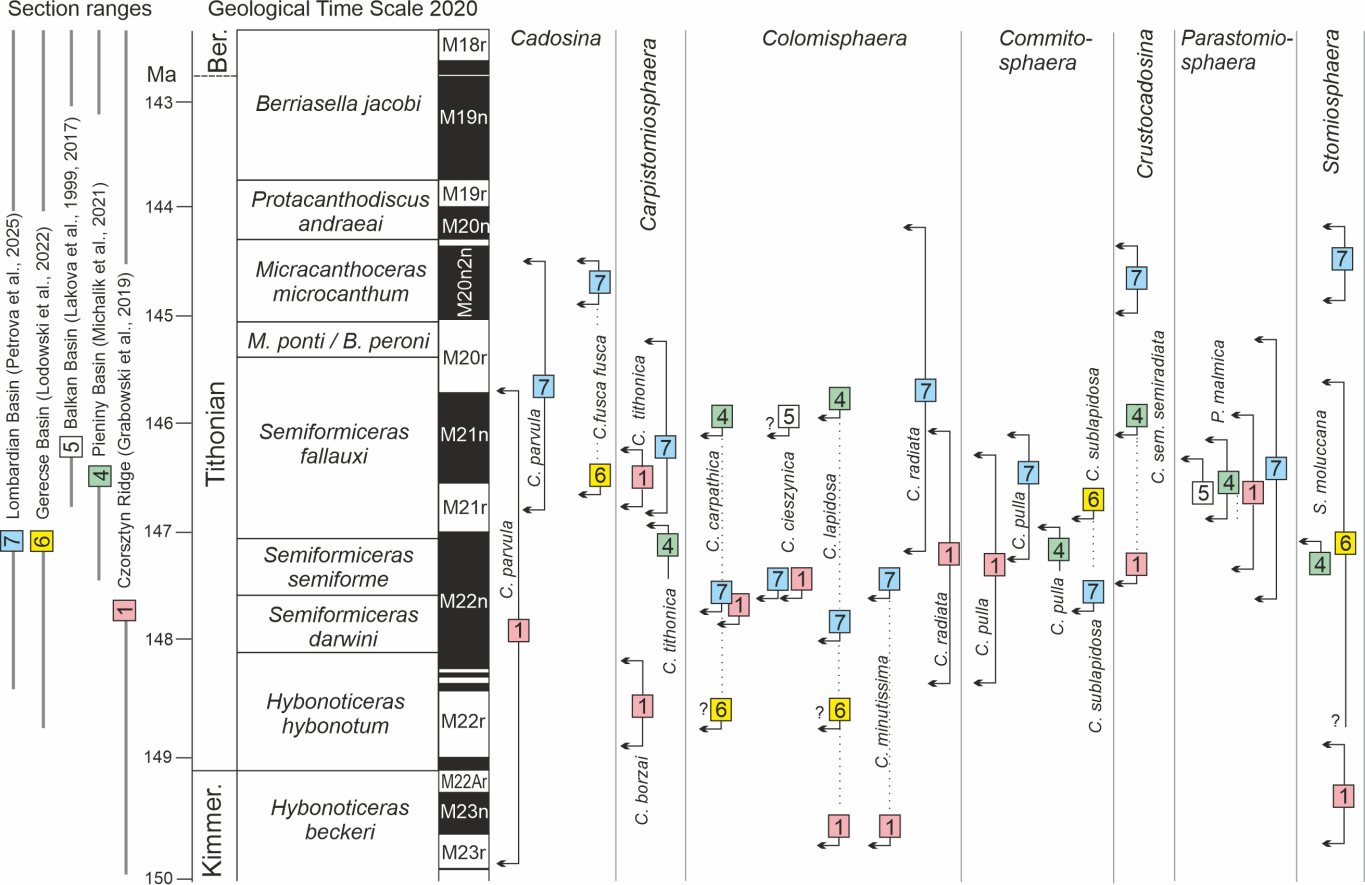
Calcareous dinocyst stratigraphic events in the uppermost Kimmerdgian and Tithonian of the Western Tethyan sections, calibrated against magnetostratigraphy and ammonite zonation (Geological Time Scale 2020). Abbreviation and location of the section symbols—see Fig. 4.

The ranges of 15 dinocyst species were compared with magnetozones M23r to M19n (Fig. 5), corresponding to the interval from the latest Kimmeridgian to the end of the Tithonian according to Hesselbo, Ogg & Ruhl (2020). For most species, the comparison focused on their FADs and LADs, while for a few long-lived taxa, only their FADs were considered. Due to the poor state of preservation of dinocysts in the Hárskút section (Lodowski et al., 2022) and the incomplete data from the Barlya section (Lakova, Stoykova & Ivanova, 1999; Lakova et al., 2017), the ranges of seven and two taxa, respectively, were used in this correlation.

**Figure 6:**
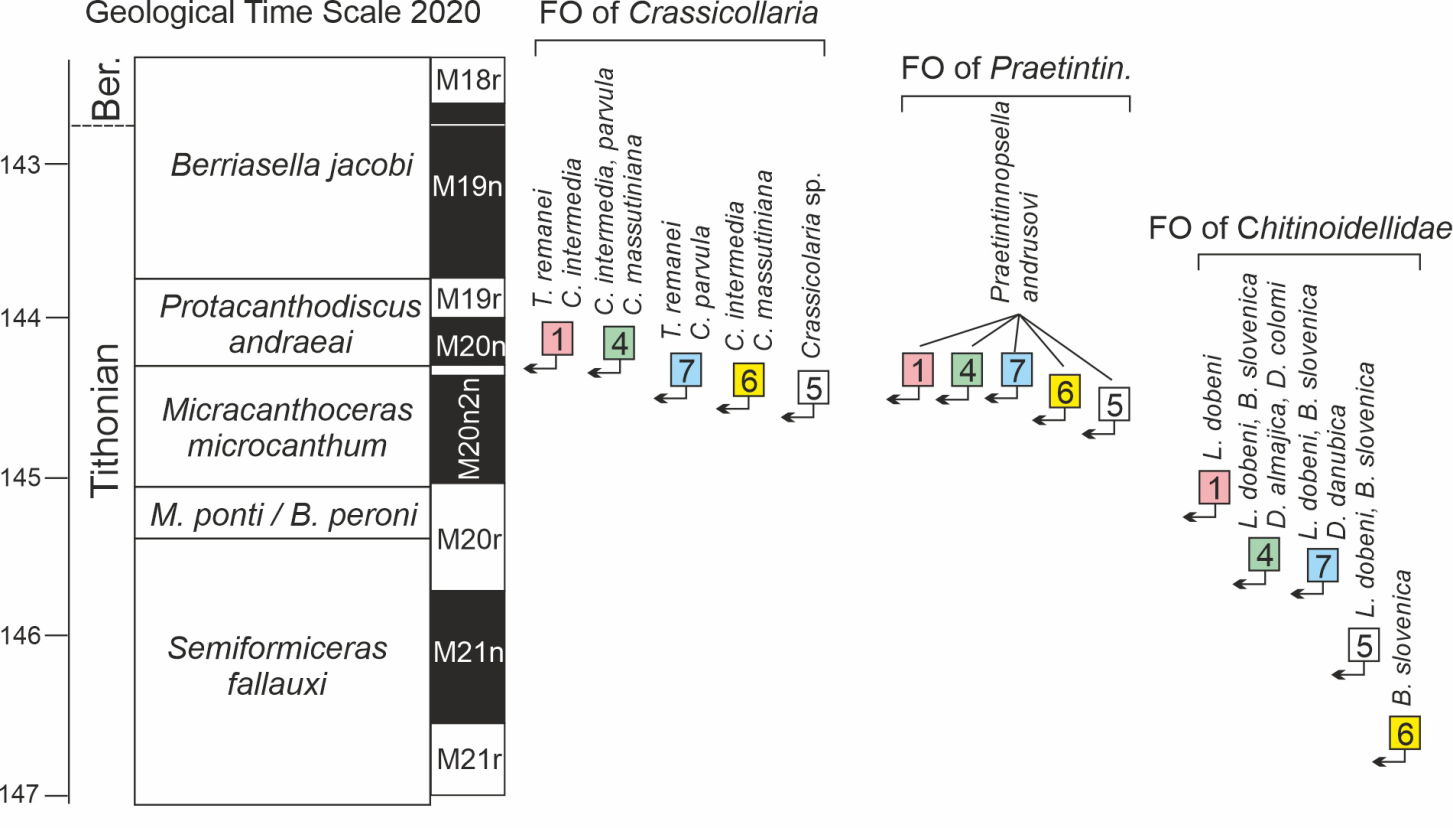
First appearances of calpionellids during the Tithonian in the Western Tethyan sections calibrated against magnetostratigraphy and ammonite zonation (Geological Time Scale 2020). Note the same trend in FAD of taxa among the analysed sections which corresponds to the geographical latitude of the sediment accumulation areas. Abbreviations of sections and their location are presented on Fig. 4.

This comparison with magnetozones demonstrates the diachroneity of the ranges of all Tethyan calcareous dinocyst species (Fig. 5). However, the degree of diachroneity varies considerably, as shown by a comparison of, for example, the ranges of *Cadosina parvula*, *Colomisphaera lapidosa*, *C. carpathica*, and *Stomiosphaera mollucana*, which exhibit strong diachroneity, *versus* the ranges of *Commitosphaera pulla* and *Parastomiosphaera malmica*, which are only moderately diachronous. The causes of this diachroneity may be related to primary environmental factors, *i.e.,* differences in physicochemical properties of seawater influencing the diversity of dinoflagellate assemblages. This is evident when comparing the FOs of species in two sections (Velykyi Kamianets and Torre de’ Bussi) that are widely separated geographically (by approximately 10 degrees of latitude; Fig. 4). Most forms from the Lombardian Basin appeared much later than those from the Czorsztyn Submerged Ridge, located at a latitude of approximately 30 N, and within a distinct palaeogeographic setting. This trend becomes particularly clear when the ranges of chitinoidellids and calpionellids are correlated with magnetostratigraphy (Fig. 6). However, for these microfossils, the degree of diachroneity of their FOs within the Western Tethys is much lower than that observed for the calcareous dinocyst species.

Differences in the stratigraphic ranges of calcareous dinocyst taxa may also result from varying degrees of sediment diagenesis, which affects both the preservation of their wall structure and the ability to identify all diagnostic taxonomic features. It is also possible that the observed pattern is influenced by the limited datasets used in this comparison, highlighting the need for closer integration of magnetostratigraphic studies with micropalaeontological research for this time interval. Regardless of the causes underlying the diachronic datum events of calcareous dinocysts, we emphasise that stratigraphic correlations of Tithonian sections based solely on calcareous dinocyst range data carry a high risk of error.

### Local dinocyst zonation and its correlation with chronostratigraphy

Correlation of Tethyan dinocyst ranges with magnetozones shows that within this assemblage, it is possible to distinguish taxa with higher stratigraphic utility, *i.e.,* those differing in their FADs. It likely applies to *Commitosphaera pulla*, *Colomisphaera radiata*, *Parastomiosphaera malmica*, *Crustocadosina semiradiata semiradiata*, and *Cadosina borzai*. It likely also applies to *Cadosinopsis rehakovae*, provided that its geographic distribution proves to be widespread. The species listed above have already served as stratigraphic markers in zonations for the upper Kimmeridgian and Tithonian in sections from the former Western Tethys and from extra-Tethyan regions.

**Figure 7:**
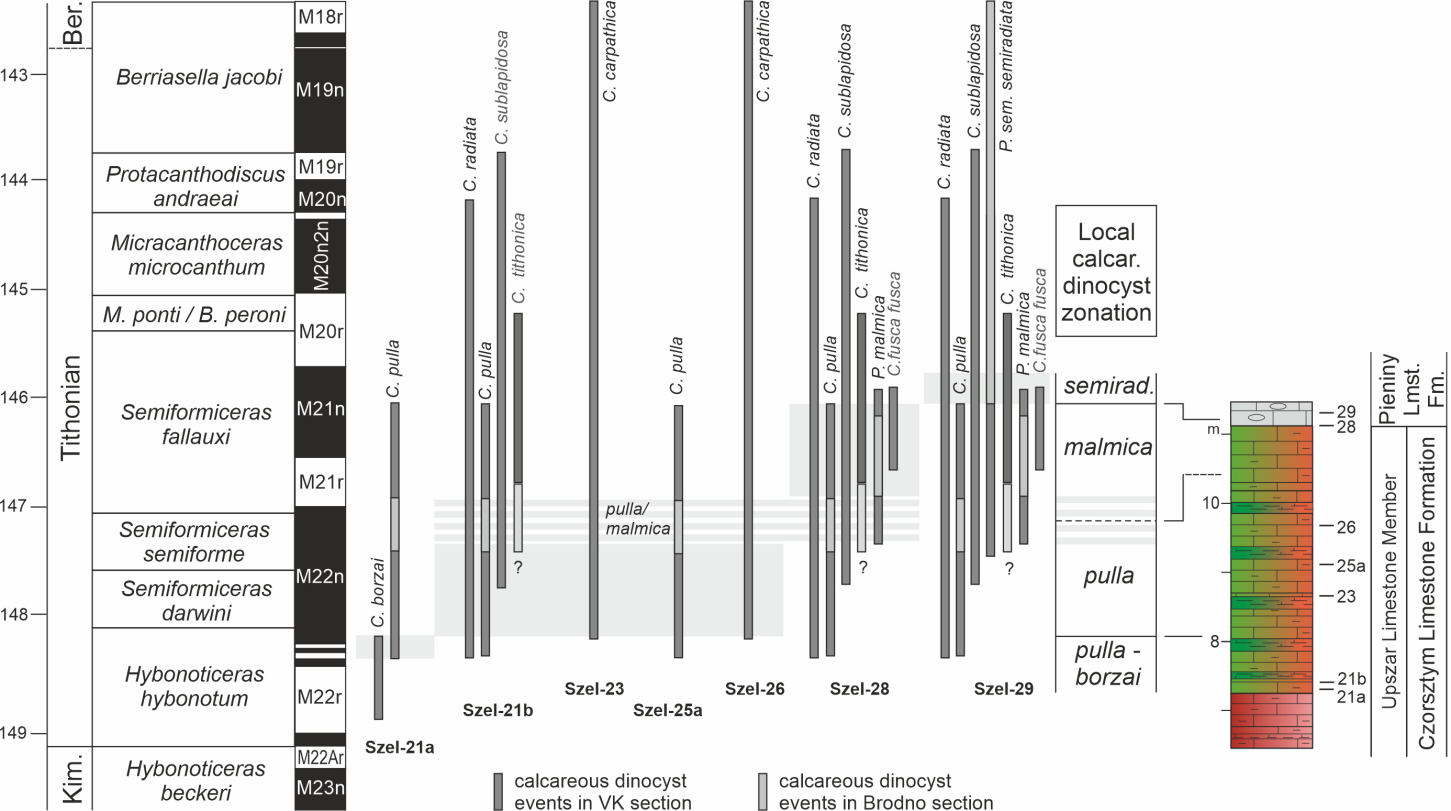
Stratigraphic ranges of selected calcareous dinocysts and the local zonation of the Szeligowy section, calibrated against magnetostratigraphy and ammonite zonation (Geological Time Scale 2020). Stratigraphic ranges refer to Velykyi Kamianets and Brodno successions (Grabowski et al., 2019; Michalík et al., 2021), deposited in the same Pieniny Basin, in which dinocyst biostratigraphy was correlated with magnetostratigraphy.

Considering that the best-established correlation between dinocyst ranges and magnetostratigraphy in the Pieniny Basin (the basin in which the sediments of the studied section were deposited) is documented in the Velykyi Kamianets (VK) and Brodno (Br) sections, and acknowledging that the studied assemblage is very similar to the assemblage from the Rogoźnik section (Reháková & Wierzbowski, 2005)—located only two kilometres from Szeligowy Creek—we assume that the ranges of species occurring in the Szeligowy section correspond closely to those documented in the VK and Br profiles. Based on this assumption, the occurrence ranges of species in successive samples of the studied succession were compared, and indicator taxa (stratigraphic markers) for the boundaries of biostratigraphic zones were selected mainly on the basis of their FADs and LADs recorded in the VK and Br profiles. These are *C. borzai*, *C. pulla*, *P. malmica*, and *C. semiradiata semiradiata* (Fig. 7).

Four dinocyst zones (Fig. 7) were distinguished within the studied Szeligowy section:

#### The *borzai–pulla* concurrent range zone

The lower boundary of this zone is not formally established in this section. The upper boundary corresponds to the LO of *C. borzai*. Ten species belonging to six genera have been identified. The quantitatively rich assemblages are dominated by *Cadosinopsis rehakovae* and *Carpistomiosphaera borzai*, accompanied by *Cadosina parvula*, *Stomiosphaera moluccana*, *Colomisphaera lapidosa*, and *C. cieszynica*, with rare occurrences of *C. minutissima*, *C. radiata*, *Comittosphaera pulla*, and *C. sublapidosa*. This zone is correlated with the lower part of the M22n magnetozone and the upper *H. hybonotum* ammonite Zone.

#### The *pulla* concurrent range zone

The lower boundary is placed at the LO of *C. borzai*, and the upper boundary corresponds to the FO of *Parastomiosphaera malmica*. Cysts of *C. pulla* are rare in this interval. The assemblages are less abundant and less diverse, comprising seven species. In addition to the species already present in the underlying zone (excluding *C. rehakovi*, *C. borzai*, *C. radiata*, and *C. sublapidosa*), *C. cieszynica* occurs in this interval. This zone is correlated with the middle and upper parts of the M22n magnetozone and with the *Semiformiceras darwini* and *S. semiforme* ammonite zones.

#### The malmica interval zone

The lower boundary of this zone is defined by the FO of *P. malmica*, whereas the upper boundary is placed at the FO of *Crustocadosina semiradiata semiradiata*. The dinocyst assemblage is slightly more abundant and similarly diverse, comprising ten species. Compared to the *pulla* zone, *Carpistomiosphaera tithonica*, *Cadosina fusca fusca*, and *P. malmica* first appear in this interval, with the latter being very abundant. The boundary between the *C. pulla* and *P. malmica* zones cannot be precisely constrained, as the FO of *P. malmica* shows a marked significant stratigraphic offset in the correlative VK and Br sections. This boundary corresponds to the transition from the M22n to the M21r magnetozone, which coincides with the transition from the *S. semiforme* to the *S. fallauxi* ammonite zone.

#### The semiradiata semiradiata interval zone

The lower boundary of this zone is defined by the FO of *C. sem. semiradiata*. The upper boundary is likewise undefined; however, it lies below the FAD of chitinoidellids, which are absent from the studied section. In the correlative VK and Br sections, the FAD of chitinoidellids is documented within the M20r magnetozone (Fig. 6). The index species is very abundant in this highly diverse assemblage, which comprise 11 species. The assemblage includes the same taxa as the *malmica* Zone, with the exception of *C. minutissima*.

Correlation of the distinguished calcareous dinocyst zones with magnetostratigraphy, based on data from the VK and Br sections, indicates that the accumulation of the variegated argillaceous limestones (Upszar Limestone Member) in the study area took place from chron M22r to at least chron M21n (Fig. 7). The transition to the accumulation of Maiolica-type sediments (Pieniny Limestone Formation) occurred in the younger part of chron M21n, *i.e.,* in the youngest part of the *P. malmica* chron. Considering the correlation of magnetozones with ammonite zonation (Hesselbo, Ogg & Ruhl, 2020) and the presence of ammonites of the “lower” Tithonian in correlative sections in the Polish part of the PKB—namely Kapuśnica 1 (Pszczółkowski & Myczyński, 2004) and Rogoźnik quarry (Reháková & Wierzbowski, 2005)—it can be stated with high probability that the calcareous dinocyst assemblage described from the studied succession corresponds to the *Hybonoticeras hybonotum* through *Semiformiceras fallauxi* zones.

## Conclusions

The stratigraphic ranges of calcareous dinocyst assemblages in the Tithonian carbonate successions of the Western Tethys exhibit pronounced diachroneity, as demonstrated by comparison with the global geomagnetic polarity timescale (GTS 2020). Calpionellid assemblages, which occur in the upper part of the Tithonian succession, show somewhat less diachroneity. This considerable diachroneity in dinocyst species ranges substantially limits the reliability of correlations between basins and even within a single large basin, especially when dinocyst assemblages constitute the only stratigraphic tool available. We emphasise that only interdisciplinary stratigraphic studies—particularly those integrating geomagnetic polarity analyses—provide a robust basis for correlating marine sections from the lower part of the Tithonian.

Among the calcareous dinocysts from the studied section, several species display stratigraphic utility related to their FAD. This allowed us to distinguish four time intervals during which the succession accumulated. Correlation of these ranges with those documented in two other sections of the Pieniny Basin (representing its shallow- and deep-water parts), themselves calibrated to magnetozones in the Velykyi Kamianets (VK) and Brodno sections (Br), made it possible to relate the dinocyst zone boundaries identified in the Szeligowy section to the global geomagnetic polarity timescale, and through it to the Tethyan ammonite zonation. Assuming that the FOs of the above-mentioned species in the studied section are comparable to those in the VK and Br sections, we infer that the accumulation of the entire studied succession lasted from polarity chron M22r to at least polarity chron M21n, corresponding to four ammonite chrons, *Hybonoticeras hybonotum* through *Semiformiceras fallauxi*. The transition to the accumulation of Maiolica-type sediments occurred in the younger part of chron M21n, *i.e.,* within the upper interval of the *Semiformiceras fallauxi* chron.

## Appendix

**Table:** 

Systematic appendix
Systematic palaeontology
Superclass **Dinoflagellata** (Bütschli, 1885) Fensome et al., 1993
Class **Dinophyceae** Pascher, 1914
Subclass **Peridiniphycidae**Fensome et al., 1993
Order **Peridiniales** Haeckel, 1894
Family **Thoracosphaeracea** Schiller, 1930
Genus ***Cadosinopsis*** Scheibner, 1967
***Cadosinopsis rehakovae***Ciurej & Bąk, 2021 (Plates 1-3A, Figs. 2–6)
Synonyms: ***Cadosinopsis rehakovii*** Ciurej and Bak, sp. nov., 2021, p. 9

## Additional Information and Declarations

### Field Study Permissions

The following information was supplied relating to field study approvals (*i.e.*, approving body and any reference numbers): The field work not required the permission.

### Data Availability

The following information was supplied regarding data availability: The data is available in Figs. 2, 3 and 7.

## References

[ref-1] Bąk M, Bąk K, Michalik M (2018). Decadal to millennial variations in water column parameters in pelagic marine environments of the Western Tethys (Carpathian realm) during Middle–Late Jurassic—evidence from the radiolarian record. Global and Planetary Change.

[ref-2] Bąk M, Chodacka S, Bąk K, Okoński S (2018). New data on the age and stratigraphic relationships of the Czajakowa Radiolarite Formation in the Pieniny Klippen Belt (Carpathians) based on the radiolarian biostratigraphy in the stratotype section. Acta Geologica Polonica.

[ref-3] Benzaggagh M, Atrops F (1996). Stratigraphic distribution of the main species of microproblematic fossils in the Upper Malm-Berriasian of the inner Pre-Rif and the Mesorif (Morocco). Biozonation and correlation with ammonites and calpionella. Comptes Rendus de L’Académie des Sciences de Paris Série IIa.

[ref-4] Benzaggagh M, Homberg C, Schnyders J, Razgallah S, Hssaida T (2015). The importance of calcareous dinoflagellate cysts and the pelagic biomicrofacies in dating upper Jurassic and lower Berriasian strata in the western Tethyan domain. Annals of Paleontology.

[ref-5] Birkenmajer K (1953). Preliminary revision of the stratigraphy of the Pieniny Klippen-Belt Series in Poland. Bulletin de L’Académie Polonaise Des Sciences, Classe 3.

[ref-6] Birkenmajer K (1962). Monuments of inanimate nature in the Pieniny Klippen Belt, part II—Klippen of Rogoźnik near Nowy Targ. Ochrona Przyrody.

[ref-7] Birkenmajer K (1963). Stratigraphy and palaeogeography of the Czorsztyn series, Pieniny Klippen Belt, Carpathians, in Poland. Studia Geologica Polonica.

[ref-8] Birkenmajer K (1977). Jurassic and Cretaceous lithostratigraphic units of the Pieniny Klippen Belt, Carpathians. Studia Geologica Polonica.

[ref-9] Birkenmajer K (1986). Stages of structural evolution of the Pieniny Klippen Belt, Carpathians. Studia Geologica Polonica.

[ref-10] Birkenmajer K, Gąsiorowski SM (1960). Stratigraphy of the Malm of the Niedzica and Branisko Series (Pieniny Klippen Belt, Carpathians) based on Aptychi. Bulletin de L’Académie Polonaise Des Sciences, Série Des Sciences Géologiques et Géographiques.

[ref-11] Birkenmajer K, Gąsiorowski SM (1961). Sedimentary character of radiolarites in the Pieniny Klippen Belt, Carpathians. Bulletin de l’Académie Polonaise des Sciences, Série des Sciences Géologiques et Géographiques.

[ref-12] Borza K (1964). The genus *Stomiosphaera* Wanner, 1940 in the Western Carpathians. Geologický Sborník.

[ref-13] Borza K (1969). The microfacies and microfossils of the Upper Jurassic and Lower Cretaceous of the cliff zone of the Western Carpathians.

[ref-14] Bown PR, Bown PR (1998). Triassic. Calcareous nannofossil biostratigraphy.

[ref-15] Ciurej A (2023). *Stomiosphaerina bakae* sp. nov., a new calcareous dinocyst of the Upper Cretaceous of the Central European Basin. PLOS ONE.

[ref-16] Ciurej A, Bąk M (2021). *Cadosinopsis rehakovii* sp. nov., a new calcareous dinocyst from the Jurassic-Cretaceous transitional interval of the Western Tethys. PLOS ONE.

[ref-17] Ciurej A, Bąk K, Bąk M (2017). Late Albian calcareous dinocysts and calcitarchs record linked to environmental changes during the final phase of OAE 1d—a case study from the Tatra Mountains, Central Western Carpathians. Geological Quarterly.

[ref-18] Ciurej A, Dubicka Z, Poberezhskyy A (2023). Calcareous dinoflagellate blooms during the Late Cretaceous ‘greenhouse’ world—a case study from western Ukraine. PeerJ.

[ref-19] Dias-Brito D (2000). Global stratigraphy, palaeobiogeography and palaeoecology of Albian–Maastrichtian pithonellid calcispheres: impact on Tethys configuration. Cretaceous Research.

[ref-20] Elbrachter M, Gottschling M, Hildebrand-Habel T, Keupp H, Kohring R, Lewis J, Sebastian Meier KJ, Montresor M, Streng M, Versteegh GJM, Willems H, Zonneveld KAF (2008). Establishing an agenda for calcareous dinoflagellate research (Thoracosphaeraceae, Dinophyceae) including a nomenclatural synopsis of generic names. Taxon.

[ref-21] Fąfara M, Dubicka Z, Niechwedowicz M, Ciurej A, Walaszczyk I (2023). Middle Campanian (Late Cretaceous) sea level rise; microfossil record of bathymetric changes. Acta Geologica Polonica.

[ref-22] Fensome RA, Taylor FJR, Norris G, Sarjeant WAS, Wharton DI, Williams GL (1993). A classification of modern and fossil dinoflagellates.

[ref-23] Fensome RA, Williams GL (2004). The Lentin and Williams Index of fossil dinoflagellates: 2004 edition.

[ref-24] Gąsiorowski SM (1962). Aptychi from the Dogger, Malm and Neocomian in the Western Carpathians and their stratigraphical value. Studia Geologica Polonica.

[ref-25] Golonka J, Bąk M, Kaminski MA, Waśkowska A (2011). Evolution of the outer carpathian basins. Integrating microfossil records from the Oceans and Epicontinental Seas.

[ref-26] Golonka J, Picha RJ, Golonka J, Picha FJ (2011). Introduction. The Carpathians and their foreland: geology and hydrocarbon resources.

[ref-27] Grabowski J, Bakhmutov V, Kdyr S, Krobicki M, Pruner P, Reháková D, Schnabl P, Stoykova K, Wierzbowski H (2019). Integrated stratigraphy and palaeoenvironmental interpretation of the Upper Kimmeridgian to Lower Berriasian pelagic sequences of the Velykyi Kamianets section (Pieniny Klipen Belt, Ukraine). Palaeogeography, Palaeoclimatology, Palaeoecology.

[ref-28] Hesselbo SP, Ogg JG, Ruhl M, Gradstein FM, Ogg JG, Schmitz MD, Ogg GM (2020). The Jurassic period. The Geologic Time Scale 2020.

[ref-29] Höll C, Zonneveld KAF, Willems H (1998). On the ecology of calcareous dinoflagellates: the Quaternary Eastern Equatorial Atlantic. Marine Micropaleontology.

[ref-30] Ivanova DK, Kietzmann DA (2017). Calcareous dinoflagellate cysts from the Tithonian-Valanginian Vaca Muerta Formation in the southern Mendoza area of the Neuquén Basin, Argentina. Journal of South American Earth Sciences.

[ref-31] Janofske D (1992). Calcareous nannofossils of the Alpine Upper Triassic.

[ref-32] Keupp H, Riding R (1991). Fossil calcareous dinoflagellate cysts. Calcareous algae and stromatolites.

[ref-33] Kietzmann DA, Encinas A (2025). Microfacies and biostratigraphy based on calpionellids and calcareous dinoflagellate cysts across the Jurassic/Cretaceous boundary, western Neuquén Basin, Baños Morales and Lo Valdés formations, Río Volcán section, Central Chile. Cretaceous Research.

[ref-34] Kietzmann DA, Iovino F, Encinas A (2022). New microbiostratigraphic data (calpionellids and calcispheres) from the Tithonian of central Chile, type section (Río Tinguiririca) of the baños del Flaco Formation. Journal of South American Earth Sciences.

[ref-35] Kietzmann DA, Llanos MPI, Iovino F (2023). Tithonian–Berriasian calcisphere (calcareous dinoflagellate cysts) zones in the Neuquén Basin, Argentina: correlation between Southern Andes and Tethyan regions. Newsletters on Stratigraphy.

[ref-36] Kietzmann DA, Scasso RA (2020). Jurassic to Cretaceous (upper Kimmeridgian–lower Berriasian) calcispheres from high palaeolatitudes on the Antarctic Peninsula: local stratigraphic significance and correlations across Southern Gondwana margin and the Tethyan realm. Palaeogeography, Palaeoclimatology, Palaeoecology.

[ref-37] Kowal-Kasprzyk J (2018). Calpionellid zones of the Tithonian–Berriasian exotic limestone clasts from the Outer Carpathians, Poland. Cretaceous Research.

[ref-38] Kutek J, Wierzbowski A (1986). A new account on the Upper Jurassic stratigraphy and ammonites of the Czorsztyn succession, Pieniny Klippen Belt, Poland. Acta Geologica Polonica.

[ref-39] Lakova I, Grabowski J, Stoykova K, Petrova S, Reháková D, Sobień K, Schnabl P (2017). Direct correlation of Tithonian/Berriasian boundary calpionellid and calcareous nannofossil events in the frame of magnetostratigraphy: new results from the West Balkan Mts, Bulgaria, and review of existing data. Geologica Balcanica.

[ref-40] Lakova I, Stoykova K, Ivanova D (1999). Calpionellid, nannofossils, and calcareous dinocyst bioevents and integrated biochronology of the Tithonian to Valanginian in the West Balcan Mountains, Bulgaria. Geologica Carpathica.

[ref-41] Lodowski DG, Pszczółkowski A, Szives O, Fozy I, Grabowski J (2022). Jurassic–Cretaceous transition in the Transdanubian Range (Hungary): integrated stratigraphy and paleomagnetic study of the Hárskút and Lókút sections. Newsletter on Stratigraphy.

[ref-42] Lodowski DG, Szives O, Virag A, Grabowski J (2024). The latest Jurassic–earliest Cretaceous climate and oceanographic changes in the Western Tethys: the Transdanubian Range (Hungary) perspective. Sedimentology.

[ref-43] López-Martínez R, Barragán R, Reháková D, Cobiella-Reguera JL (2013). Calpionellid distribution and microfacies across the Jurassic/Cretaceous boundary in western Cuba (Sierra de los Órganos). Geologica Carpathica.

[ref-44] Matejová M, Reháková D, Aubrecht R, Ledvényiová L, Měchová L (2022). Unusual microfacies character of the Pieniny Limestone in the Orava sector of the Pieniny Klippen Belt. Acta Geologica Slovaca.

[ref-45] Michalík J (1994). Notes on the paleogeography and paleotectonics of the Western Carpathian area during the Mesozoic. Mitteilungen der Österreichischen Geographischen Gesellschaft.

[ref-46] Michalík J, Grabowski J, Lintnerová O, Reháková D, Kdýr S, Schnabl P (2021). Jurassic–Cretaceous boundary record in Carpathian sedimentary sequences. Cretaceous Research.

[ref-47] Michalík J, Reháková D, Grabowski J, Lintnerová O, Svobodová A, Kdýr S, Schnabl P (2016). Stratigraphy, plankton communities, and magnetic proxies at the Jurassic/Cretaceous boundary in the Pieniny Klippen Belt (Western Carpathians, Slovakia). Geologica Carpathica.

[ref-48] Montresor M, Montesarchio E, Marino D, Zingone A (1994). Calcareous dinoflagellate cysts in marine sediments of the Gulf of Naples (Meditenanean Sea). Review of Palaeobotany and Palynology.

[ref-49] Myczyński R (1973). Middle Jurassic stratigraphy of the Branisko succession in the vicinity of Czorsztyn (Pieniny Klippen Belt, Carpathians). Studia Geologica Polonica.

[ref-50] Nowak W (1968). Stomiosphaerids of the Cieszyn beds (Kimeridgian–Hauterivian) in the Polish Cieszyn Silesia and Their Stratigraphical Value, [English Summary]. Annales de la Société Géologique de Pologne.

[ref-51] Nowak W (1974). Stomiosphaerina nov. gen. (incertae sedis) of the Upper Cretaceous in the Polish Flysch Carpathians. Annales de la Société Géologique de Pologne.

[ref-52] Nowak W (1976). *Parastomiosphaera malmica* (Borza) from the Polish Carpathians and their stratigraphical value for the Lower Tithonian deposits. Annales de la Société Géologique de Pologne.

[ref-53] Petrova S, Reháková D, Erba E, Grabowski J (2025). Tithonian–Berriasian calpionellid and calcareous dinocyst biostratigraphy, and microfacies in the Torre de’ Busi section (Lombardian Basin, northern Italy). Cretaceous Research.

[ref-54] Plašienka D (2003). Dynamics of Mesozoic pre-orogenic rifting in the Western Carpathians. Mitteilungen der Österreichischen Geographischen Gesellschaft.

[ref-55] Prokešova R, Plašienka D, Milovský R (2012). Structural pattern and emplacement mechanisms of the Križna cover nappe (Central Western Carpathians). Geologica Carpathica.

[ref-56] Pszczółkowski A (2009). The Tithonian–earliest Berriasian *Nannoconus* zones in selected sections of the Pieniny Klippen Belt and the Western Tatra Mountains (southern Poland). Studia Geologica Polonica.

[ref-57] Pszczółkowski A, Myczyński R (2004). Ammonite-supported microfossil and nannoconid stratigraphy of the Tithonian–Hauterivian limestones in selected sections of the Branisko Succession, Pieniny Klippen Belt (Poland). Studia Geologica Polonica.

[ref-58] Pszczółkowski A, Myczyński R (2010). Tithonian–Early Valanginian evolution of deposition along the proto-Caribbean margin of North America recorded in Guaniguanico successions (western Cuba). Journal of South American Earth Sciences.

[ref-59] Reháková D (2000a). Evolution and distribution of the Late Jurassic and Early Cretaceous calcareous dinoflagellates recorded in the Western Carpathian pelagic carbonate facies. Mineralia Slovaca.

[ref-60] Reháková D (2000b). Calcareous dinoflagellate and calpionellid bio-events *versus* sea-level fluctuations recorded in the West-Carpathian (Late Jurassic/Early Cretaceous) pelagic environments. Geologica Carpathica.

[ref-61] Reháková D, Matyja BA, Wierzbowski A, Schlögl J, Krobicki M, Barski M (2011). Stratigraphy and microfacies of the Jurassic and lowermost Cretaceous of the Veliky Kamenets section (Pieniny Klippen Belt, Carpathians, western Ukraine). Volumina Jurassica.

[ref-62] Reháková D, Rožič B (2019). Calpionellid biostratigraphy and sedimentation of the Biancone limestone from the Rudnica Anticlinale (Sava Folds, eastern Slovenia). Geologija.

[ref-63] Reháková D, Wierzbowski A (2005). Microfacies and stratigraphic position of the Upper Jurassic Rogoża coquinas at Rogoźnik, Pieniny Klippen Belt, Carpathians. Volumina Jurassica.

[ref-64] Řehánek J (1982). New species of the genus *Colomisphaera* Nowak from the Tithonian and Upper Cretaceous. Geologicky Sbornik–Geologica Carpathica.

[ref-65] Řehánek J (1992). Valuable species of cadosinids and stomiosphaerids for determination of the Jurassic-Cretaceous boundary (vertical distribution, biozonation). Scripta Geology.

[ref-66] Řehánek J, Cecca F (1993). Calcareous dinoflagellate cysts biostratigraphy in Upper Kimmeridgian-Lower Tithonian pelagic limestones of Marches Apennines. Revue de Micropaléontologie.

[ref-67] Ruffo Rey L, Kietzmann DA, Bressan GB (2018). Calcispheres of the Vaca Muerta Formation (Tithonian) in the Covunco stream section, Neuquén Province. Journal of the Argentine Geological Association.

[ref-68] Sarti C (1999). Protancyloceras (Ammonoidea) in the Lower Tithonian sequences of the Trento Plateau (Venetian Alps, Northern Italy). Profil.

[ref-69] Schmid MS, Fügenschuh B, Kounov A, Maţenco L, Nievergelt P, Oberhänsli R, Pleuger J, Schefer S, Schuster R, Tomljenović B, Ustaszewski K, Van Hinsbergen DJJ (2020). Tectonic units of the Alpine collision zone between Eastern Alps and western Turkey. Gondwana Research.

[ref-70] Stampfli GM, Hochard C, Murphy JB, Keppie JD, Hynes AJ (2009). Plate tectonics of the Alpine realm. Ancient orogens and modern analogues.

[ref-71] Tangen K, Brand LE, Blackwelder PL, Guillard RRL (1982). *Thoracosphaera heimii* (Lohmann) Kamptner is a dinophyte: observations on its morphology and life cycle. Marine Micropaleontology.

[ref-72] Vašíček Z, Michalík J, Reháková D (1994). Early Cretaceous stratigraphy, palaeogeography and life in Western Carpathians.

[ref-73] Vink A, Zonneveld KAF, Willems H (2000). Distribution of calcareous dinoflagellate cysts in surface sediments of the western subtropical Atlantic Ocean, and their potential use in palaeoceanography. Marine Micropaleontology.

[ref-74] Vishnevskaya VS (2017). The Jurassic-Cretaceous boundary in Boreal Russia: radiolarian and calcareous dinoflagellate potential biomarkers. Geological Quarterly.

[ref-75] Wendler JE, Gräfe KU, Willems H (2002). Reconstruction of mid-Cenomanian orbitally forced palaeoenvironmental changes based on calcareous dinoflagellate cysts. Palaeogeography, Palaeoclimatology, Palaeoecology.

[ref-76] Wierzbowski A, Pallini S (1990). The taxonomy and phylogenetic significance of Early Tithonian ammonites of the genus *Protancyloceras* Spath from the Pieniny Klippen Belt (Carpathians, Poland). Atti II Conv. Intern. Fossili, Evoluzione, Ambiente, Pergola 1987.

[ref-77] Zonnevald KAF, Brune A, Willems H (2000). Spatial distribution of calcareous dinoflagellate cysts in surface sediments of the Atlantic Ocean between 13°N and 36°S. Review of Palaeobotany and Palynology.

[ref-78] Zügel P (1994). Distribution of calcareous dinoflagellate cysts in the Cenomanian/ Turonian of Western France and Northern Germany. Courier Forschungsinstitut Senckenberg.

